# Retraction Note: Mesenchymal stem cell-derived exosomes exert ameliorative effects in type 2 diabetes by improving hepatic glucose and lipid metabolism via enhancing autophagy

**DOI:** 10.1186/s13287-022-03191-6

**Published:** 2022-10-20

**Authors:** Qin He, Lingshu Wang, Ruxing Zhao, Fei Yan, Sha Sha, Chen Cui, Jia Song, Huiqing Hu, Xinghong Guo, Mengmeng Yang, Yixin Cui, Yujing Sun, Zheng Sun, Fuqiang Liu, Ming Dong, Xinguo Hou, Li Chen

**Affiliations:** 1 Department of Endocrinology, Qilu Hospital of Shandong University, No. 107 Wenhua Xi Road, Jinan, 250012 Shandong China; 2 Institute of Endocrine and Metabolic Diseases of Shandong University, Jinan, 250012 Shandong China; 3 Key Laboratory of Endocrine and Metabolic Diseases, Shandong Province Medicine & Health, Jinan, 250012 Shandong China

## Retraction Note: Stem Cell Research & Therapy (2020) 11:223 https://doi.org/10.1186/s13287-020-01731-6

The Editors-in-Chief have retracted this article because of the following concerns:

- Figure 2I: overlapping regions are present within this image
- Figure 3D: overlapping regions are present within this image
- Figure 5A: this image is incorrect
- Figure 5E and 6H: overlapping regions are present within and between these images

The Editors-in-Chief therefore no longer have confidence in the data presented.

Authors Xinghong Guo and Xinguo Hou agree with this retraction but disagree with the wording of the retraction notice. All other authors agree to this retraction.

